# Influence of spatial structure migration of overlying strata on water storage of underground reservoir in coal mine

**DOI:** 10.1371/journal.pone.0292357

**Published:** 2024-01-29

**Authors:** Zhiguo Cao, Suolin Jing, Lujun Wang, Fanbao Meng, Yanning Han

**Affiliations:** 1 State Key Laboratory of Water Resource Protection and Utilization in Coal Mining, Beijing, China; 2 College of Energy and Mining Engineering, Shandong University of Science and Technology, Qingdao, China; 3 College of Mechanical and Electronic Engineering, Shandong University of Science and Technology, Qingdao, China; 4 The First Exploration Team of Shandong Coalfield Geologic Bureau, Qingdao, China; University of Science and Technology Beijing, CHINA

## Abstract

Underground reservoir technology for coal mines can realize the coordinated development of coal exploitation and water protection in water-shortage-prone areas. The seepage effect of the floor seriously affects the safety of underground reservoirs under the action of mining damage and seepage pressure. Focusing on the problem of floor seepage in underground reservoirs, a spatial mechanical model of underground reservoirs was established. The main factors affecting the seepage of the surrounding rock were studied. The seepage pressure law in different stages of spatial structure evolution of overlying strata was explored. The results showed that pressure change was the main factor affecting the stability of a reservoir’s surrounding rock. The pore space between the broken and fractured rock in the water-flowing fractured zone was the main water storage space, which was directly related to the development of a breaking arch. According to the spatial structure evolution process of the overlying strata, the water storage state of an underground reservoir was divided into two stages and three situations. The seepage pressure was mainly affected by the water pressure and the overlying strata weight. The water pressure was affected by the reservoir head height, and the overlying strata weight was mainly affected by the overlying strata thickness.

## 1. Introduction

The northwest region is one of the main production areas of coal resources in China. The production accounts for about 70% of the national output. However, water resources account for only 3.9%, resulting in very serious water shortages for mines [1]. Underground reservoir technology uses coal mine goaf to realize water source storage and circulation reuse [2, 3]. There are still many scientific problems to be overcome in underground reservoir technology [4], such as the occurrence of seepage in the floor that seriously affects the safety of underground reservoirs.

The seepage characteristics of fractured rock mass directly affect the stability of the floor. After the underground reservoir stores water, the pore water pressure in the floor increases, damaging the original joints of the surrounding rock. Many scholars have carried out relevant research on the stability and failure of underground reservoirs in coal mines. Gu studied the dynamic response and stability of coal pillars under earthquake action and believed that the floor of the mine was also damaged by the earthquake [5]. Li Jianhua believed that the soaking effect had little effect on the mechanical properties of coal samples, which were mainly affected by the bedding structure. Therefore, the influencing factors affecting floor seepage are mainly its structure, external stresses and osmotic pressure [6]. Bai et al. studied the limit water head of a coal pillar in an underground reservoir and obtained the relationship between the stability of a reservoir and the critical value of the bearing water pressure of a coal pillar [7]. Huo et al. pointed out that the stress distribution and transmission of the floor are superimposed by a coal pillar, and the damage to the floor is very serious when the disturbance occurs again [8]. Zhu et al. found that only when the elastic part of the coal pillar was greater than 31% of the overall coal pillar would the coal pillar be in a long-term stable state. The process of seepage formation is the result of the combined action of stress and seepage [9]. Terzaghi proposed the basic theory and initial model of seepage and profit coupling in geotechnical media [10]. Witherspoon first defined the stress change caused by seepage as a “coupling effect” and proposed a related theory [11]. Noorishad improved the coupling theory [12]. At the same time, international scholars have also used different test methods to verify the inverse correlation between the permeability coefficient and effective confining pressure [13–15]. Herda simulated the seepage of fractured rock mass and expounded the path of seepage [16]. Yao et al. studied the mining-induced seepage strain mechanism of the floor and found that the mining disturbance made the permeability coefficient of the surrounding rock change significantly [17]. Ma et al. [18] and Li et al. [19] established a seepage model of a mined-out area floor and calculated the permeability characteristics of the floor strata through the relationship between the stress and permeability characteristics. Wang et al. revealed the coupling mechanism of rock stress–seepage and simulated and studied the stress state and seepage characteristics of floor mining above confined water [20]. The research shows that the permeability of the floor is closely related to horizontal stress. Wang et al. pointed out that the coal seam floor was alternately damaged as mining progressed, and a plastic zone and large channel were formed under the combined action of mining and confined water pressure, resulting in a sudden change in permeability [21]. The above studies pointed the stress–seepage coupling law of the floor from the perspective of confined water inrush on the floor, but it cannot be effectively applied to the seepage control for the floor of an underground reservoir. Some researchers have studied the impermeability of floor rock. Guo et al. carried out in situ compression–seepage tests of different lithological structures and obtained the impermeability of the original state of the rock strata with different lithological structures and concluded that the impermeability of the floor rock strata depends on the original structural conditions and fissure properties of the rock strata, which are not related to the lithology of the rock strata itself [22]. Huang et al. tested the floor aquifuge through a high-pressure water pressure drilling test. The research shows that there is a positive correlation between the permeability coefficient and pressure water flow [23]. Shao et al. [24], Jiang et al. [25] and Zhang et al. [26] also conducted similar studies. The above research explores the main factors affecting the permeability coefficient of the floor. The results show that the permeability coefficient is affected by water pressure. The change in the permeability coefficient under changing water pressure is studied, and the mechanical characteristics of the rock layer under the action of the seepage field and stress field are not fully considered.

Floor water inrush belongs to floor confined water inrush, and the water storage state of underground reservoir in coal mine is different from it. The difference between groundwater reservoir floor seepage and confined water floor water inrush is mainly reflected in two aspects. First, the floor confined water mainly comes from Ordovician limestone karst water, while the main coal seam in northwest China was formed in the late Jurassic period, so the water sources of the two are different from the stratigraphic age. Secondly, the main force source of reservoir floor seepage is the water pressure generated by the height of water level or the resultant force of water pressure and overlying strata gravity, while the force source of water inrush from the floor above confined water is the pressure given by the stratum to the confined aquifer. At the same time, both will be affected by primary or mining disturbance cracks. The water in the coal mine underground reservoir exists freely in the goaf, and the water body may also be affected by the movement of the overlying strata.

Because The research of underground reservoir is biased towards the black box problem, the most intuitive is the change of external surrounding rock. Relatively mature application of transfer rock beam theory and overlying strata spatial structure can measure part of the parameter values, but still can not be fully quantified. Therefore, based on this theory, qualitative and semi-quantitative analysis of the stability of underground reservoirs was conducted in different time periods under different working conditions, in order to obtain results through subjective evaluation and measurement of phenomena. Based on the mature spatial structure evolution of overburden rock, this paper deduces the change of seepage pressure inside underground reservoir. In view of this, this paper actively carries out research on the occurrence characteristics of seepage in the surrounding rock of underground reservoirs and explores the relationship between seepage pressure and overlying strata movement and structure, which has an important engineering value for studying the seepage of the floor and determining the reasonable reservoir water storage.

## 2. Model construction

Under long-term external disturbance, an underground reservoir is prone to floor damage, coal pillar collapse, etc., resulting in the overall destruction of the reservoir [27]. Rock failure under stress–seepage coupling is a typical problem, which is different from rock failure under single action [28, 29]. Underground reservoirs have particular importance because of their special geographical environment. Firstly, the floor is affected by mining, forming fracture damage zones with certain depths [30]. Secondly, the floor experiences secondary damage under the coupling of stress and seepage, wherein the strength is further reduced, and the overall stability of the reservoir is reduced [31].

**Fig 1:**
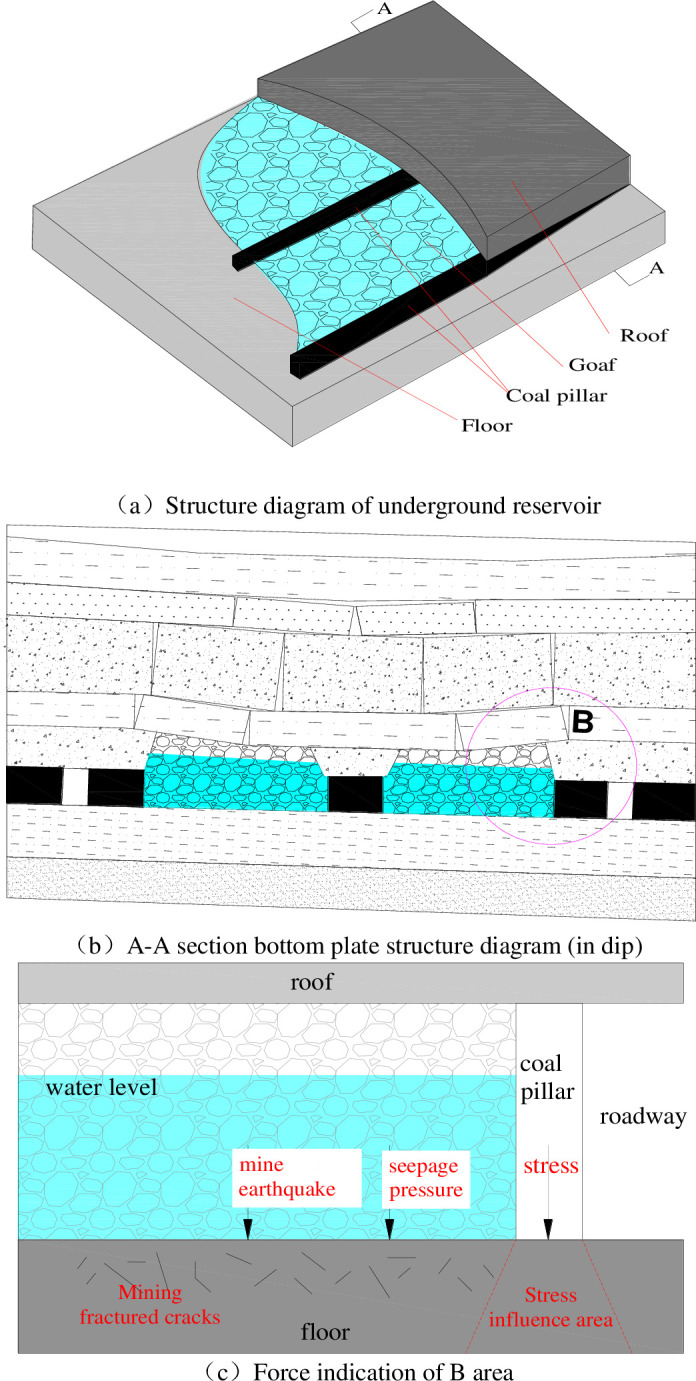
The spatial mechanics model of surrounding rock in underground reservoir. (a) Structure diagram of underground reservoir. (b) A-A section bottom plate structure diagram (in dip). (c) Force indication of B area.

A coal mine underground reservoir in Shendong mining area is selected as the research object. The geometric shape, size and material properties of the underground reservoir are collected. It is determined that the reservoir is mainly composed of multiple goafs as the main water storage space, the boundary coal pillar of the mining area is the water retaining dam, and the roof and floor in the mining area are the boundaries. According to the theory of transfer rock beam, the stress environment of the model and the spatial structure of the rock mass around the underground reservoir are determined. The analysis shows that the underground reservoir will be in different stable states at different stages of overlying strata movement. The initial conditions and excavation methods of the underground reservoir model are the basic conditions for the formation of goaf after coal seam mining. The model is a combination of multiple goafs formed by the natural caving method, forming a relatively closed goaf composed of coal pillars and roof and floor surrounding rock. Considering the influence of overlying strata movement on the stability of underground reservoir, based on the influence of stress and water pressure on stability of underground reservoir, the spatial mechanical model of surrounding rock of underground reservoir is established by studying the water seepage in damaged rock mass and the failure phenomenon of rock mass under the water action, as shown in Fig 1.

Considering the change in pressure as the main factor of surrounding rock stability design, the influence of different seepage pressures and stresses on the seepage of an underground reservoir floor is studied. The sudden change in the permeability coefficient is key to the seepage instability of the surrounding rock in an underground reservoir. The starting pressure gradient is negatively correlated with the permeability coefficient [32]. The occurrence of floor seepage in an underground reservoir is affected by the rock mass permeability coefficient and external seepage pressure. The permeability coefficient of the floor is related to its porosity and the stress distribution and transmission of the floor. At the same time, the seepage of the floor is affected by water pressure.

The seepage characteristics of the surrounding rock of the underground reservoir are mainly affected by factors such as seepage pressure, coal pillar stress and mining-induced fracture, and the possibility of earthquakes caused by mining disturbance [33]. According to statistics, the degree of influence of each factor is seepage pressure>pillar stress>mining-induced fracture>mine earthquake. During the operation of an underground reservoir, seepage pressure and coal pillar stress are the main factors affecting the stability of the surrounding rock. Under the action of seepage pressure, water flows into the floor where the fracture zone has been formed, causing secondary damage to the floor. The coal pillar stress changes the flow characteristics of water in the rock. In the case of the reservoir group, the instability of the reservoir roof and floor will directly lead to the water level overrunning the lower reservoir, which will affect the stability of the surrounding rock of the lower reservoir.

On this basis, the relationship between seepage pressure and overlying strata failure and water head height is studied, and the distribution of floor seepage pressure in different stages of overlying strata spatial structure evolution is explored.

## 3. Factors of floor seepage based on Darcy’s law

The research shows that the mutability of the permeability coefficient and the joint structure of the surrounding rock medium itself are the two main factors affecting groundwater flow [34]. According to the analysis of the spatial model of the underground reservoir, the mutation of the permeability coefficient is mainly due to the influence of the non-uniform stress affecting the floor and the cracks related to mining damage. At the same time, the change in the seepage pressure will also cause secondary damage to the damaged floor, thus affecting the permeability coefficient of the floor.

Darcy’s law describes the linear relationship between the seepage velocity of the water and the hydraulic gradient in a saturated media [35]. The seepage flow is positively correlated with the cross-sectional area of the specimen and the difference between the upper and lower heads and negatively correlated with the length of the seepage path, as follows.

$$Q=kA\frac{h_{2}-h_{1}}{\Delta L}$$ (1)

where Q is the seepage flow, k is the permeability coefficient, A is the cross-sectional area of the specimen, Δ*L* is the length of the seepage path, and *h*_2_、*h*_1_ are the upper and lower water heads, respectively.

At the same time, the product of the flow rate and the cross-sectional area is equal to the seepage flow, as follows.

Q=Av (2)

$$v=kJ=k\frac{\Delta h}{\Delta L}$$ (3)

where J is the hydraulic gradient, $J=\frac{\Delta h}{\Delta L}$. Extending Darcy’s law to a three-dimensional case, the differential form of a three-dimensional Darcy ’s law is obtained, as follows.


$$v_{x}=-k_{x}\frac{\partial h}{\partial x},\;v_{y}=-k_{y}\frac{\partial h}{\partial y},\;v_{z}=-k_{z}\frac{\partial h}{\partial z}$$ (4)

Whether it is a saturated medium or an unsaturated medium, the occurrence of medium seepage must obey the law of the conservation of mass. Large numbers of experimental studies have shown that Darcy’s law is still applicable to the seepage of fluid in the unsaturated zone of the medium. Based on this theory, the saturated zone and unsaturated zone of the seepage medium are regarded as a unified continuous medium, and a unified equation is used to describe the seepage field [36, 37]. In this paper, two variables of water pressure and overlying strata gravity (external stress of medium) are introduced into Darcy’s law formula and deduced. The continuity equation is the basic equation to study groundwater movement. Based on the continuity equation and mass conservation equation, the differential equation of groundwater movement suitable for an underground reservoir is established.

**Fig 2:**
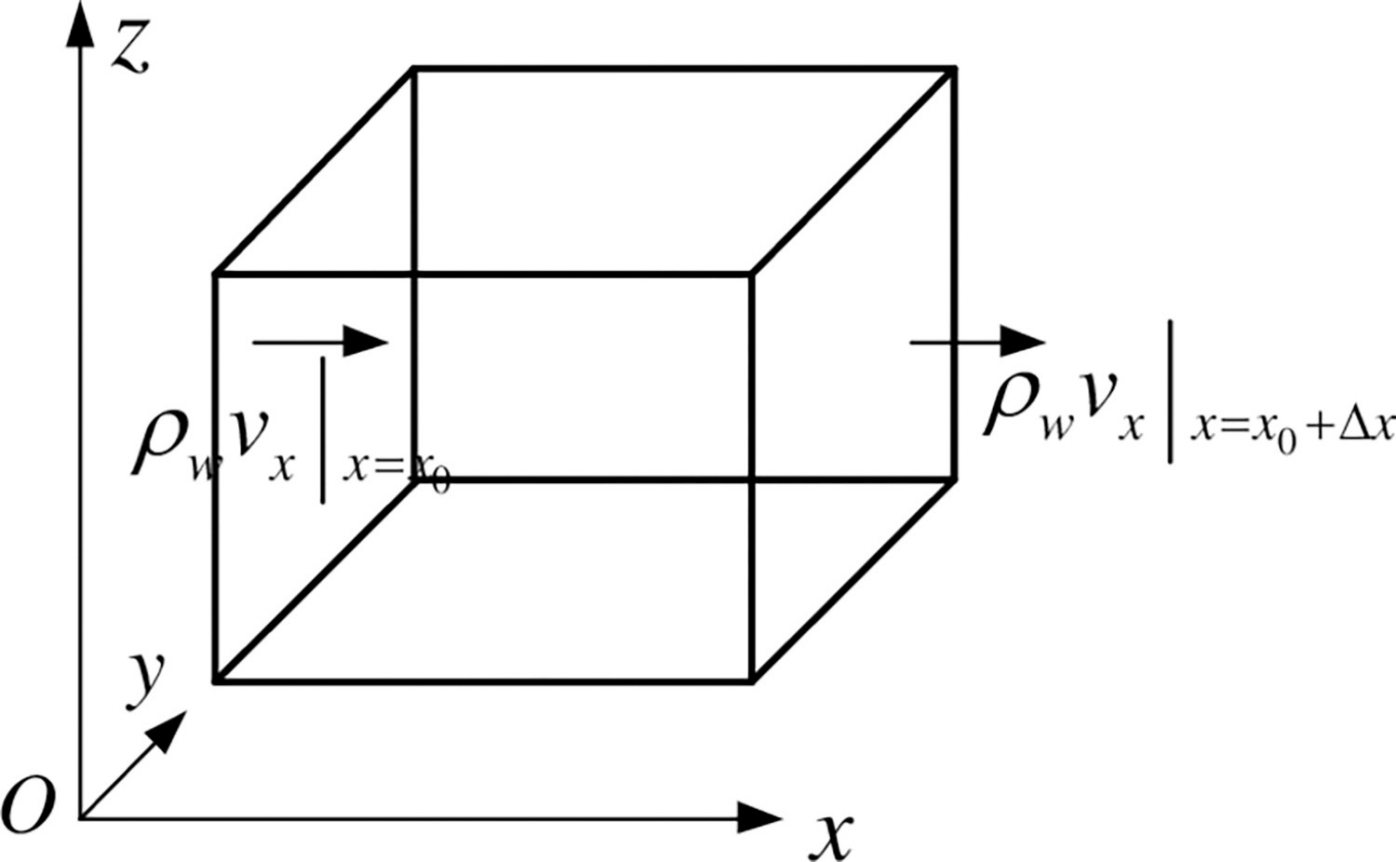
Microelement control unit.

A micro-element control unit (Fig 2) is established, assuming that the water is compressible [38], the solid particles cannot be compressed, the porous medium skeleton is compressible in the vertical direction *Z*, and Δ*x*, Δ*y* is a constant. Therefore, only fluid density *ρ*_*w*_, porosity *ϕ* and unit height Δ*z* change with pressure.

According to the law of conservation of mass, the difference between the inflow mass and outflow mass per unit of time at a certain point is equal to the changing mass in the micro-element. The continuity equation is as follows.

$$-[\frac{\partial (\rho_{w}v_{x})}{\partial x}+\frac{\partial (\rho_{w}v_{y})}{\partial y}+\frac{\partial (\rho_{w}v_{z})}{\partial z}]\Delta x\Delta y\Delta z=\frac{\partial (\rho_{w}\phi \Delta z)}{\partial t}\Delta x\Delta y$$ (5)

where *ρ*_*w*_ is the fluid density, *ϕ* is the medium’s porosity, *C* is the saturation, and *v*_*i*_ is the velocity component in a certain direction.

In the case of considering the seepage pressure, the change state of water and the porous media under seepage pressure are considered. It is known that the equation of the state of water under osmotic pressure is as follows.

$$\frac{d\rho_{w}}{dp}=\beta_{w}\rho_{w}$$ (6)

where *p* is the seepage pressure, which includes the water pressure and overlying strata gravity, and *β*_*w*_ is the compression coefficient of the water. In general, water can be regarded as incompressible, and the variation in water density in this paper is small enough that it is considered to be constant.

When there is pressure outside of the medium, the compressibility of the medium is reflected in the changing pore volume. The influence of rock compressibility on the seepage process is mainly reflected in two aspects: on the one hand, the change in seepage pressure causes a change in the pore size of the seepage medium, namely porosity, which is a function of pressure. On the other hand, the change in porosity causes a change in permeability. Therefore, the compression coefficient is used to represent the relationship between porosity and osmotic pressure. It is known that the state equation of a porous media under seepage pressure is as follows.

$$\frac{de}{dp}=\alpha_{b}(1+e)$$ (7)

where *e* is the void ratio and *α*_*b*_ is the volume elastic compression coefficient of a porous media. The change in porosity of water-bearing strata under an external force is proportional to the increment in pressure.

It is known that seepage pressure is the main factor affecting the seepage of the reservoir floor, which includes two parts: water pressure and gravity acting on the overlying strata. Its differential form is as follows.

$$\begin{array}{l} dp_{w}=\rho_{w}gdh\\ d\sigma_{z}=\gamma dH \end{array}\}$$ (8)

where *p*_*w*_ is the water pressure, *σ*_*z*_ is the gravity acting on the overlying strata, g is a constant, h is the water head, *γ* is the volumetric weight of the overlying strata, and *H* is the buried depth of the coal seam.

According to the above formula, the seepage continuity equation is transformed into a seepage control equation of the seepage floor of an underground reservoir with water pressure and overlying strata gravity as control variables.

When seepage conforms to Darcy’s law, the three-dimensional Darcy’s law of Eq 4 can be substituted into the continuity equation of Eq 5 to obtain.


$$[\frac{\partial }{\partial x}(\rho_{w}k_{x}\frac{\partial h}{\partial x})+\frac{\partial }{\partial y}(\rho_{w}k_{y}\frac{\partial h}{\partial y})+\frac{\partial }{\partial z}(\rho_{w}k_{z}\frac{\partial h}{\partial z})]\Delta z=\frac{\partial (\rho_{w}\phi \Delta z)}{\partial t}$$ (9)

Combining Eq 8 with Eq 6:

$$\frac{\partial \rho_{w}}{\partial t}=\frac{\partial \rho_{w}}{\partial p}\frac{\partial p}{\partial t}=\frac{\partial \rho_{w}}{\partial p}\frac{\partial (p_{w}+\sigma_{z})}{\partial t}=\beta_{w}\rho_{w}(\rho_{w}g\frac{\partial h}{\partial t}+\gamma \frac{\partial H}{\partial t})$$ (10)

Combining Eq 8 with Eq 7:

$$\begin{array}{l} \frac{\partial e}{\partial t}=\frac{\partial e}{\partial p}\frac{\partial p}{\partial t}=\frac{\partial e}{\partial p}\frac{\partial (p_{w}+\sigma_{z})}{\partial t}=\alpha_{b}(1+e)(\rho_{w}g\frac{\partial h}{\partial t}+\gamma \frac{\partial H}{\partial t})\\ \phi =\frac{e}{1+e}\\ \frac{\partial \phi }{\partial t}=\frac{\partial }{\partial t}(\frac{e}{1+e})=\frac{1}{1+e}(\frac{\partial e}{\partial t}-\phi \frac{\partial e}{\partial t}) \end{array}\}$$ (11)

Therefore, the right side of Formula (9) can be changed to:

$$\begin{array}{l} \frac{\partial (\rho_{w}\phi \Delta z)}{\partial t}=\frac{\Delta z}{1+e}[e\beta_{w}\rho_{w}(\rho_{w}g\frac{\partial h}{\partial t}+\gamma \frac{\partial H}{\partial t})+\rho_{w}(\frac{\partial e}{\partial t}-\phi \frac{\partial e}{\partial t})]\\ \;=\frac{\Delta z}{1+e}(\rho_{w}g\frac{\partial h}{\partial t}+\gamma \frac{\partial H}{\partial t})[e\beta_{w}\rho_{w}+\rho_{w}\alpha_{b}(1-\phi )(1+e)]\\ \;=\Delta z\rho_{w}(\phi \beta_{w}+(1-\phi )\alpha_{b})(\rho_{w}g\frac{\partial h}{\partial t}+\gamma \frac{\partial H}{\partial t}) \end{array}$$ (12)

The seepage continuity equation of an underground reservoir floor is simplified as follows.


$$\frac{\partial }{\partial x}(k_{x}\frac{\partial h}{\partial x})+\frac{\partial }{\partial y}(k_{y}\frac{\partial h}{\partial y})+\frac{\partial }{\partial z}(k_{z}\frac{\partial h}{\partial z})=(\phi \beta_{w}+(1-\phi )\alpha_{b})(\rho_{w}g\frac{\partial h}{\partial t}+\gamma \frac{\partial H}{\partial t})$$ (13)

In the case of a certain permeability coefficient, water pressure and overlying strata gravity changes will directly affect the seepage velocity, thus affecting the water seepage behaviour. The water pressure is mainly affected by the water head height, and the overlying strata gravity is mainly affected by the thickness of the overburdened rock.

## 4. Results and discussion

Based on the spatial structure model of the overlying strata in the stope, the relationship between seepage pressure and the mode of the overlying strata is studied, and the variation law of seepage pressure under different stages of overlying strata failure is explored.

### 4.1. Spatial structure evolution of overlying strata

**Fig 3:**
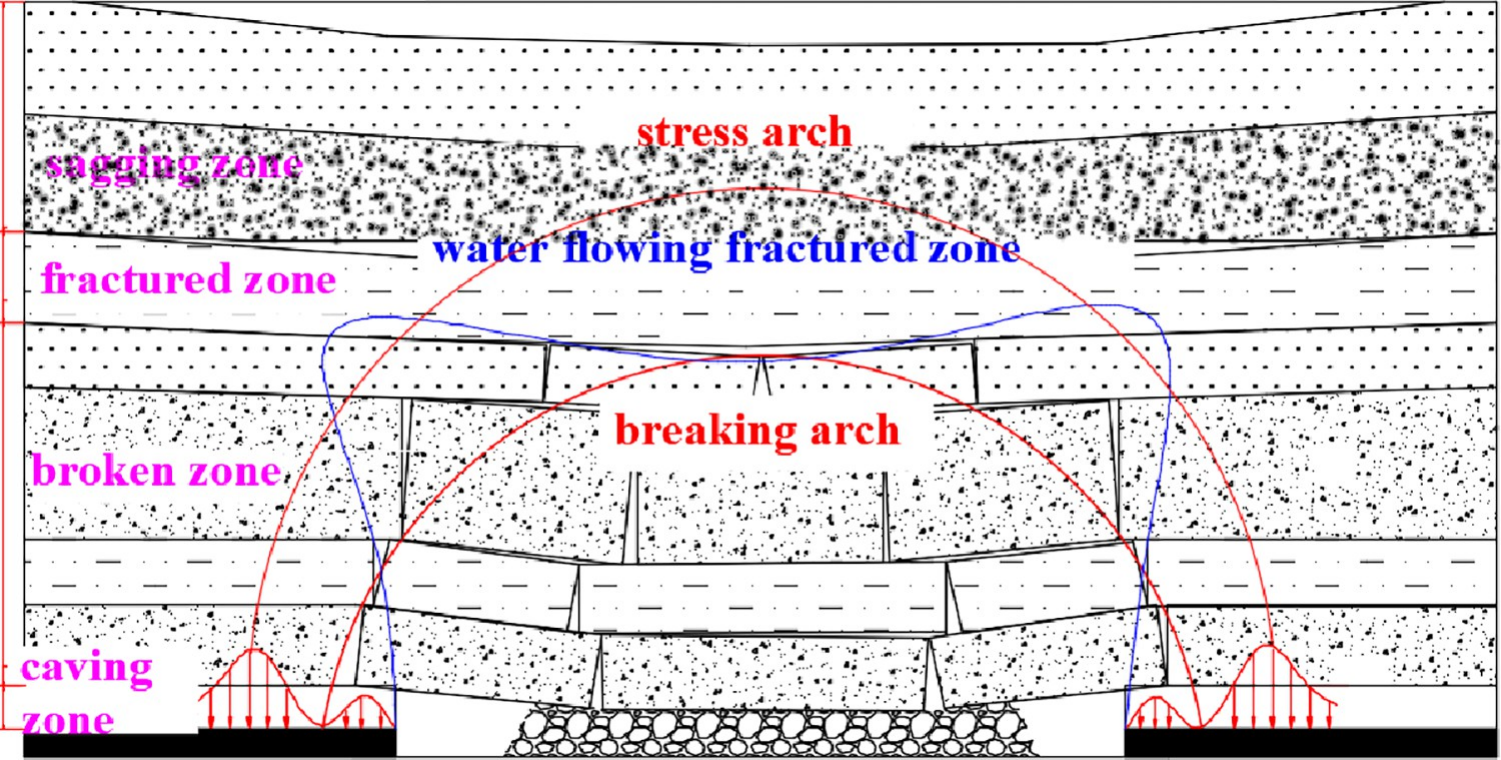
Spatial structure of overlying strata in stope.

With the advance of the working face, the overlying strata are broken in turn from bottom to top, and a breaking arch and stress arch begin to form [39]. The moving strata, which have a direct influence on the stress, are the main component of the spatial structure of the overlying strata. The spatial structure model of the overlying strata is established (Fig 3) [40].

According to the existing theory, the overlying strata can be divided into four zones in the vertical direction [41]. The failure form of rocks in a caving zone is mainly breakup, and that in the fracture zone is mainly fracture. The rock strata in the broken zone are composed of a series of ’transfer rock beams’ that move simultaneously (or almost simultaneously) and can always maintain the connection of transfer force in the advancing direction. The breaking arch consists of the caving zone and the water-flowing fractured zone, and the height of the latter is basically consistent with the range of the breaking arch [42]. The main water storage space is the water-flowing fractured zone [43].

When the mining conditions (working face width, mining thickness, buried depth, overlying strata properties) are certain, the height of the breaking arch increases with the increase in the working face’s advancing distance. The structural development process is divided into two stages: insufficient mining and sufficient mining [44]. The formation of a breaking arch is gradually formed with mining. The overlying strata experience a process of suspension–collapse–compaction. In the early stage of the formation of a breaking arch, the overburden structure is mainly manifested as the fracture and hinge of a beam. After the mining of a working face, the rock strata under the breaking arch undergo a compaction stage.

**Fig 4:**
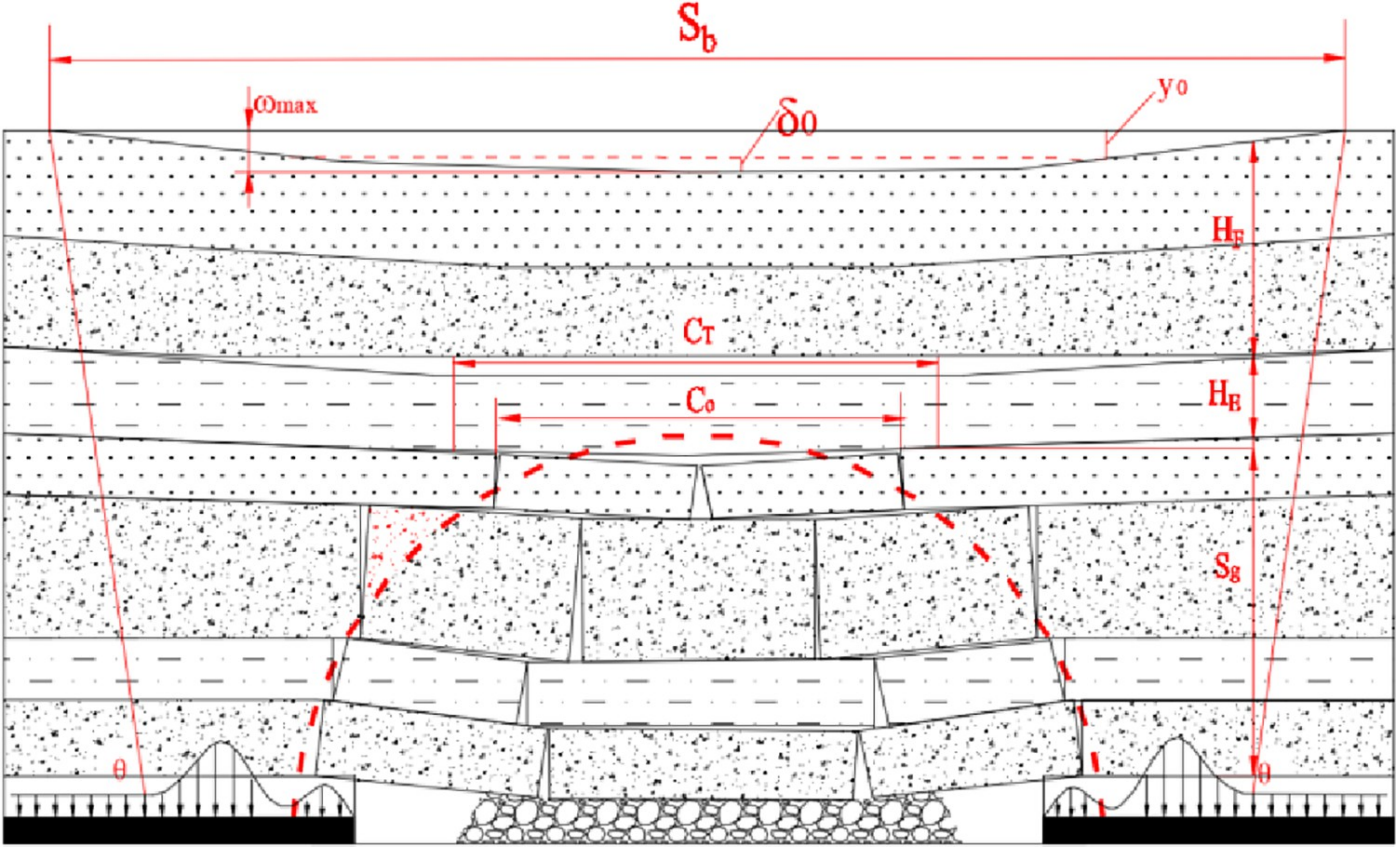
Mining subsidence prediction model.

The prediction and control model of mining subsidence based on the correlation between the length of mining face and the fracture step distance of overlying strata is put forward in Fig 4 [42]. The surface subsidence can be calculated by the working condition, and the overburden structure can be calculated by the surface subsidence to judge the stage of overburden migration.

### 4.2. Seepage pressure based on spatial structure of overlying strata

**Fig 5:**
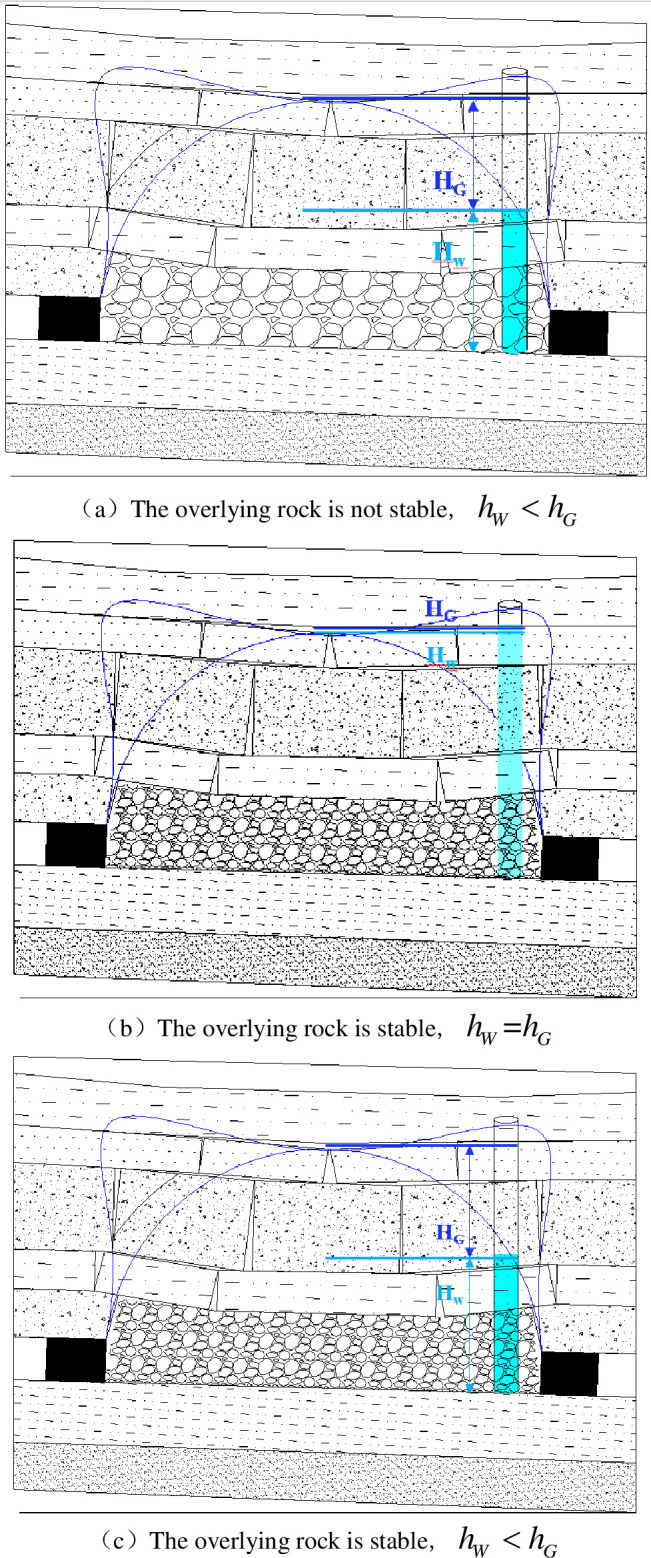
The relationship between reservoir water head and overlying strata structure. (a) The overlying rock is not stable, *h*_*W*_<*h*_*G*_. (b) The overlying rock is stable, *h*_*W*_ = *h*_*G*_. (c) The overlying rock is stable, *h*_*W*_<*h*_*G*_.

Pressure is the main factor affecting the seepage of an underground reservoir floor [45]. The seepage pressure of an underground reservoir comes in two aspects. On the one hand, it is the water pressure dominated by the change in the water head. The second is the overlying strata weight, which is dominated by the thickness of that participating in the movement. According to the process of the overlying strata’s spatial structure evolution, the water storage state of an underground reservoir is divided into two stages, as shown in Fig 5.

The first stage: the breaking arch began to form, but the overlying strata in the gob had not been compacted and stabilized. The interior of the underground reservoir is not completely sealed at this stage. Partial fracture space in the water-flowing fractured zone filled with water. The water body has not yet contacted the bottom of the complete aquiclude above the water-flowing fractured zone and can flow. As shown in Fig 5(A), the height of the water head is less than that of the water-flowing fractured zone, and the seepage pressure is only determined by the water head height.

$$p=\rho_{w}gh_{w}$$ (14)

where *h*_*w*_ is the height water head, m. The seepage pressure is only related to the height of the water head until the water body fills the water-flowing fractured zone.

The second stage: the breaking arch fully formed, the overlying strata have collapsed and compacted stability. At the same time, according to whether the water body is fully filled, the range of water-flowing fractured zone is divided into two cases.

①Water is filled completely (Fig 5(B)). Water pressure is related to water head height and overlying strata gravity. When the water is filled completely with the range of the water flowing fractured zone, there will be some pressure inside the reservoir. At this time, the seepage pressure is not only from the water pressure produced by the water head height, but also from the stress of overlying strata on the water body.


$$p=\rho_{w}gh_{G}+\gamma (H_{E}+H_{F})$$ (15)

②Water body has not yet filled the water flowing fractured zone, and the water body has a free surface (Fig 5(C)). Water pressure is related to head height.

With the condition of stable compaction of overlying strata, the reservoir forms a closed space. When the water-flowing fractured zone is not fully filled, the condition of seepage pressure is similar to the first stage. What is more, due to the overall sealing of the reservoir, there is a certain gas pressure inside the reservoir. The factors affecting the internal gas pressure of the reservoir are mainly the porosity of the surrounding rock of the reservoir and are also affected by the size of the reservoir space. At this time, the seepage pressure is composed of water pressure and gas pressure generated by its own water body.



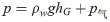

(16)


Above all, the influence of seepage pressure on reservoir floor seepage is different under different conditions. The maximum water level of an underground reservoir can be obtained by clarifying the seepage pressure and composition of a reservoir under different overlying strata structures, which is conducive to controlling the safe and stable operation of an underground reservoir.

### 4.3. Influence of overlying strata failure mode on seepage

The practical mine pressure theory points out that the form of movement and failure of overlying strata on the goaf determines the law of mine pressure, which directly affects the pressure of roof on gangue and floor. There are two basic forms of overlying strata failure: bending failure and shear failure.

①Movement form of bending failure.

**Fig 6:**
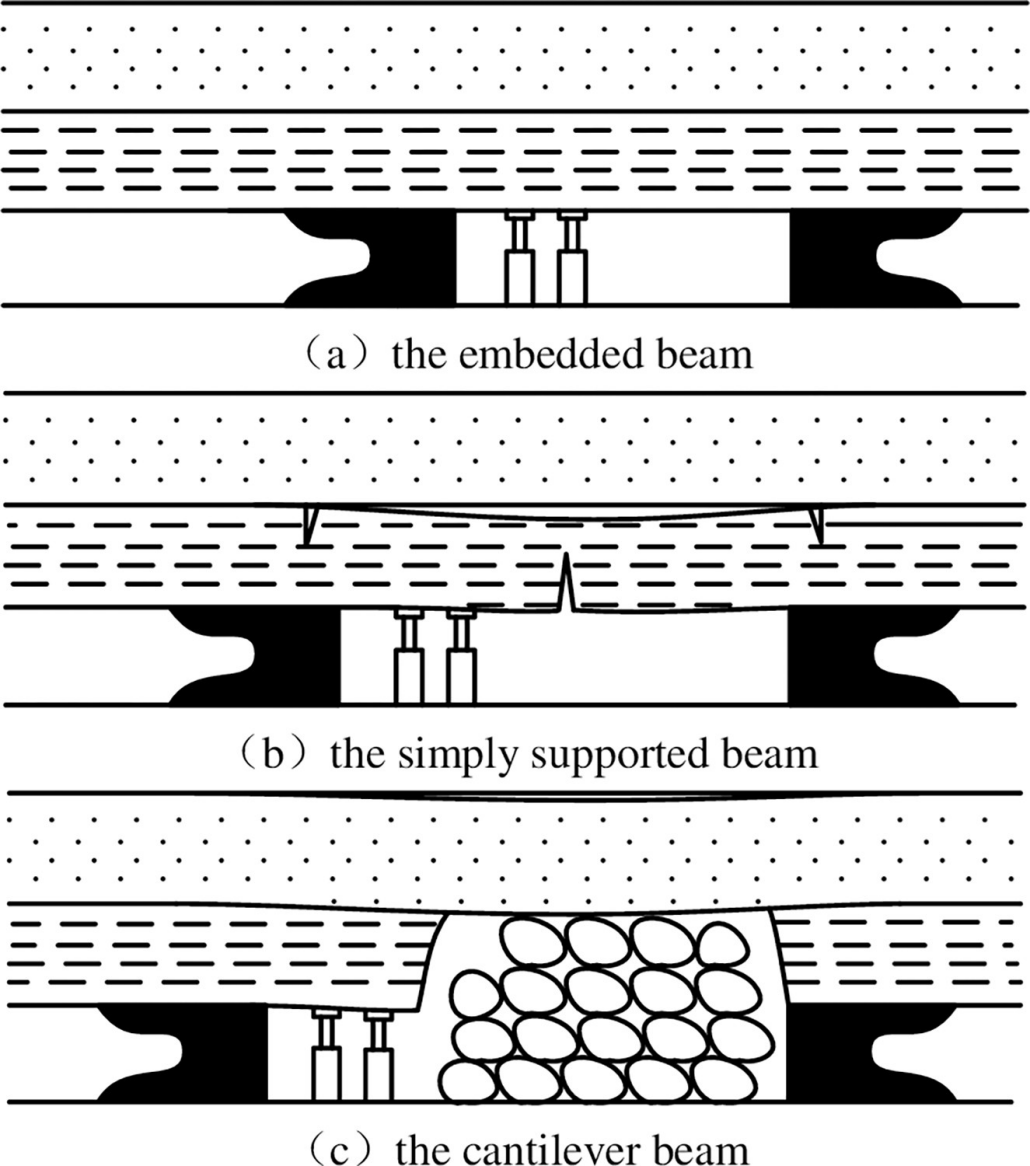
Movement stage of rock bending failure. (a) the embedded beam. (b) the simply supported beam. (c) the cantilever beam.

As the mining face advances, after the bending settlement develops to a certain limit, the bending tensile stress exceeds the strength of the rock layer itself. The two ends of the embedded beam (Fig 6(A)) crack to form a simply supported beam (Fig 6(B)), and the middle crack collapses to form a cantilever beam (Fig 6(C)), as shown in Fig 6.

②Movement form of shear failure.

**Fig 7:**
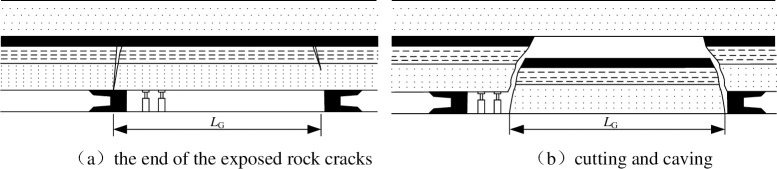
Movement stage of shear failure. (a) the end of the exposed rock cracks. (b) cutting and caving.

After the rock stratum is exposed, a small bending deformation occurs, and the end of the exposed rock stratum is cracked. In the case of no cracking in the middle of the rock stratum, the sudden overall cutting and caving is shown in the Fig 7.

The different movement forms of overburden failure have a significant impact on the water storage of underground reservoirs. Firstly, the available space of goaf under bending failure is larger than that under shear failure. Secondly, under the shear failure situation, the stress and action range of the gangue in the goaf on the floor are greater than the bending failure.

It is assumed that the compaction degree of gangue in goaf is *k*_1_ and the contact ratio is *k*_2_, which affect the influence of seepage pressure and stress on the floor respectively. The *k*_1_ directly affects the permeability coefficient, and the two are negatively correlated. In the case of a certain gravity of the overlying strata, the greater the *k*_2_, the more uniform the stress transfer. Due to the opaque characteristics inside the underground reservoir, the quantitative relationship between them needs further study.

### 4.4. Influence of other factors on seepage

In addition to the above factors, the factors that may affect the stability of underground reservoirs may include.

①abutment pressure

**Fig 8:**
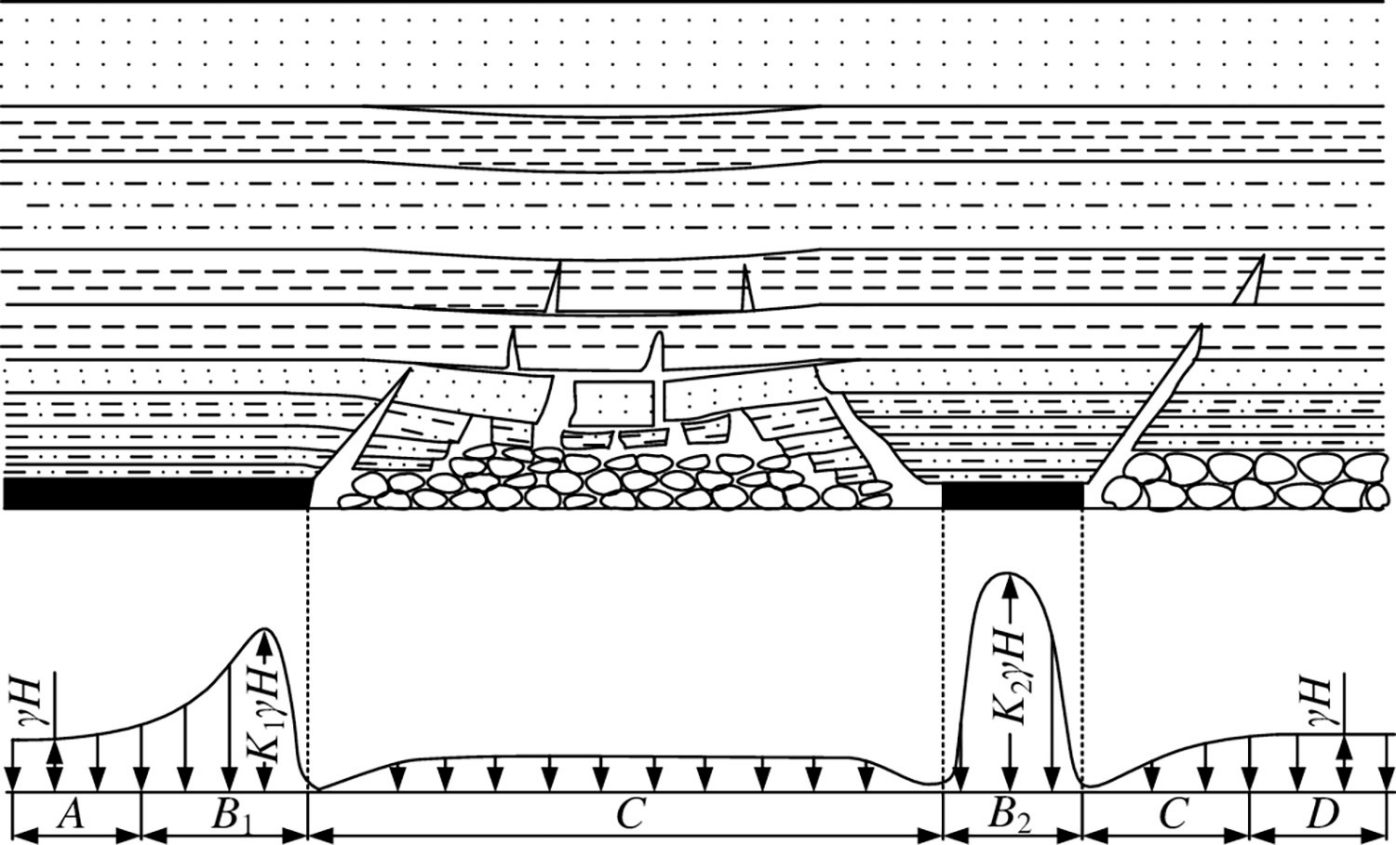
The stress distribution of the mined area and its adjacent coal pillars, A is original rock stress area; B is stress increasing area; C is stress reduction zone; D is the stress stable region.

The abutment pressure is affected by the stress of the original rock, the shape and size of the goaf, the properties and dynamics of the overlying strata in the goaf, the strength of the coal pillar and its surrounding mining conditions, and the mining thickness of the coal seam. The distribution parameters of the abutment pressure are mainly obtained by field measurement. The distribution of abutment pressure in stope is shown in Fig 8.

②Softening effect of water on rock

The study of rock mechanics shows that the strength of saturated water rock, original humidity rock and dry rock is different. The strength of saturated water rock is the lowest, especially the weak rock in sedimentary strata is more affected by water content. Some weak rocks even collapse and lose strength after soaking in water. The floor strata of underground reservoir are often in saturated water state.

③The erosion of water

There are micro-scale and macro-scale small discontinuities such as pores, joints and cracks in rock mass, and there are also larger fault planes and sedimentary planes. The erosion of water makes the original small discontinuous surface of rock mass change.

## 5 Conclusions

Based on the spatial model of an underground reservoir and seepage theory, the seepage law of a floor at different stages of overlying strata structure evolution was explored.

A spatial mechanical model of an underground reservoir was established. It is concluded that the seepage pressure, coal pillar stress and mining-induced fracture were the factors affecting the seepage of the floor

The seepage control equation for an underground reservoir floor was derived with water pressure and overlying strata weight as variables. The seepage of a reservoir floor was mainly affected by the change in seepage pressure.

The correlation between the underground seepage pressure and the structural evolution of the overlying strata was studied, and the influence of the movement and failure stage of the overlying strata on the seepage pressure and reservoir capacity was explored. The variation law of seepage pressure under three kinds of overlying strata structure evolution in the two stages of overlying strata failure was obtained. The seepage pressure was mainly affected by the height of the water head and the weight of the overlying strata.
